# Diagnosing Endometrial Carcinoma in a Patient With Atrophic Endometrium and Postmenopausal Bleeding

**DOI:** 10.7759/cureus.27939

**Published:** 2022-08-12

**Authors:** Madhumitha Prabhakaran, Shagun Tuli, Anju Beesetty

**Affiliations:** 1 Obstetrics and Gynecology, Saveetha Medical College and Hospital, Chennai, IND; 2 Obstetrics and Gynecology, University of Global Health Equity, Kigali, RWA; 3 General Medicine, Soochow University, Suzhou, CHN

**Keywords:** atrophic endometrium, evaluation of postmenopausal bleeding, serous endometrial carcinoma, outpatient hysteroscopy, endometrial biopsy, endometrial carcinoma, transvaginal sonography, postmenopausal bleeding

## Abstract

Endometrial carcinoma is the leading cause of gynecologic malignancies in the United States. Unlike other malignancies, endometrial carcinoma presents early with the most common clinical symptom being uterine bleeding (including irregular menses, inter-menstrual bleeding, and postmenopausal bleeding, or PMB). Hence, the evaluation of PMB should have efficient and effective strategies to prevent a missed diagnosis of malignancy and to facilitate an early diagnosis for potentially curative treatment.

Transvaginal ultrasound is appropriate to evaluate PMB initially. If imaging reveals an endometrial thickness of ≤4 mm, endometrial sampling is not warranted, given the high negative predictive value (>99%) of this finding for endometrial carcinoma. In women with persistent or recurrent bleeding, if blind endometrial sampling does not show endometrial hyperplasia or malignancy, further testing with hysteroscopy with dilation and curettage is indicated. However, in cases of PMB with an endometrial thickness of ≤4 mm on transvaginal ultrasound, little information can be gained from endometrial sampling alone as the chance of getting an adequate sample is low and malignancy is rare. For such patients, outpatient hysteroscopy has become a convenient and cost-effective procedure that may be done before an endometrial sampling.

## Introduction

Postmenopausal bleeding (PMB) is defined as bleeding per vaginam occurring a year or more after the last menstrual period. There are many etiologies for PMB, ranging from benign causes like atrophic endometrium to malignant neoplasms, making it essential to diagnose it correctly. The prevalence of PMB is approximately 10%, of which 10% cases account for endometrial carcinoma while benign endometrial polyps account for another 20%-40% [1]. The most common cause of PMB is atrophic endometrium or vagina. Other causes include cervicitis, cervical polyp, carcinoma of the cervix, and carcinoma of the vagina. Risk factors for the development of endometrial carcinoma include thickened endometrium, polycystic ovarian syndrome, obesity, long duration of menopause, estrogen therapy, Tamoxifen, diabetes, hypertension, and older age [2].

Unlike other gynecologic malignancies, endometrial cancer presents earlier with clinical symptoms and provides a chance of curative treatment. Approximately 90% of endometrial cancer cases present with PMB [3]. More than 70% of cases of endometrial cancer are stage I at the time of diagnosis, with a reported five-year survival rate of 90% [4]. With an increase in time to diagnosis and treatment, survival decreases with higher staging and poorer differentiation. Hence, it is prudent to make an accurate and timely diagnosis with appropriate investigations. All cases of PMB must be investigated further to rule out endometrial carcinoma and hyperplasia. Current evaluation methods include transvaginal ultrasound, hysteroscopy, and endometrial sampling [5].

## Case presentation

**Figure 1:**
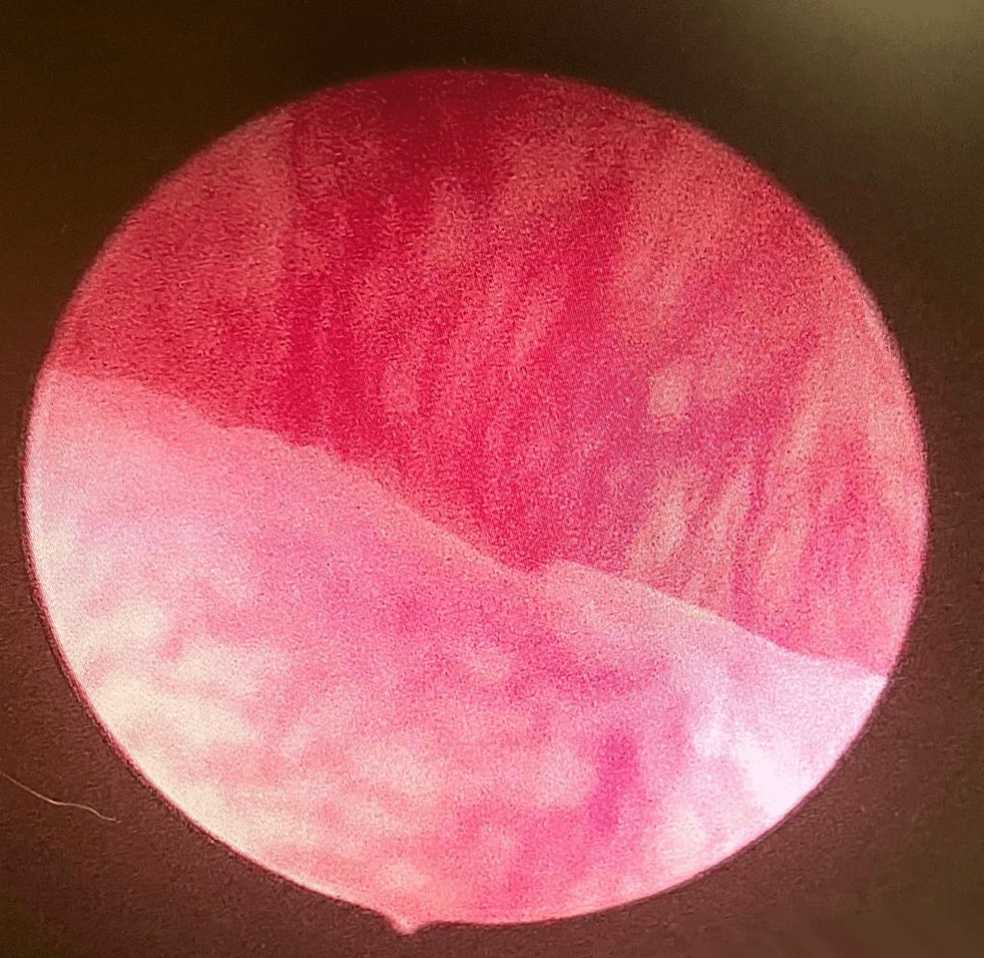
Hysteroscopic image of a highly vascular polypoid lesion

A 54-year-old woman with an obstetric score of gravida 2 para 2 presented with complaints of two episodes of spotting per vaginam one week apart. She attained menopause at the age of 51. She had two cesarean deliveries and she did not have any significant past medical history. She was up-to-date with cervical cancer screening and never had an abnormal pap smear. She was non-alcoholic, non-smoker, and sexually active with one male partner. Her body mass index was 27 and her vitals were normal. On speculum examination, the vulva, vagina, and cervix appeared normal. Transvaginal ultrasound revealed an endometrial thickness of 4 mm, multiple fibroids with the largest one being 5 cm in diameter, and no adnexal masses or free fluid. In view of the postmenopausal bleeding, an outpatient endometrial sampling was obtained that showed scant stripes of benign endometrial tissue consistent with atrophic endometrium. Two weeks later, she experienced another episode of vaginal spotting. A hysteroscopy was performed, which picked up a highly vascular polypoid lesion of 0.5 x 0.4 x 0.3 cm in size (Figure 1). The diseased tissue was sent for biopsy, which revealed serous endometrial carcinoma, overexpression of p53 and p16, and increased mitotic activity. The patient was managed with a hysterectomy and bilateral salpingo-oophorectomy for stage 1B endometrial carcinoma. She recovered without complications and is currently in remission.

## Discussion

American College of Obstetrics and Gynecologists (ACOG) Opinion No. 734 stipulates that a transvaginal ultrasound is appropriate to evaluate PMB initially. If the image reveals an endometrial thickness of ≤4 mm, there is a 99% negative predictive value for endometrial cancer. Furthermore, co-existing conditions like obesity, myomas, adenomyosis, and previous uterine surgery can cause difficulty in ultrasound visualization of the uterus [3]. Our patient had an endometrial lining of 4 mm, making malignancy extremely rare. But she had a history of cesarean deliveries and uterine myomas, which might have distorted the uterine shape preventing proper visualization of the endometrium on ultrasound.

According to ACOG Opinion No. 734, in women with persistent or recurrent bleeding, if the blind endometrial sampling does not show endometrial hyperplasia or malignancy, further testing with hysteroscopy is indicated [3]. A study done by Elsandabesee and Greenwood showed that performing an endometrial sampling avoided the need for hysteroscopy in 61.5% of cases with an endometrial thickness of greater than 4 mm. But there is only a 27% chance of obtaining an adequate endometrial sample when the endometrial thickness is less than 5 mm [6]. Further review of data from 13 published studies showed that an endometrial thickness of 5 mm or less found by ultrasonography had a sensitivity of 90% and a specificity of 54% for the detection of endometrial cancer as opposed to 98% and 35%, respectively, with a reduced cutoff of 3 mm [4]. This indicates a possibility of a missed diagnosis with decreased detection of a true negative. The patient under discussion had an endometrial lining of 4 mm, which may not have provided an adequate sample, hence hysteroscopy could aid in early diagnosis rather than endometrial sampling.

A study done by Cooper et al. concluded that the initial evaluation of PMB using transvaginal ultrasound with a 4 mm cut-off should be restricted to women with risk factors like high body mass index (>30), diabetes, or nulliparity. In women without risk factors, office hysteroscopy may be more cost-effective and convenient as they are not required to go to the operating room [7]. Given the complaints of PMB despite a thin endometrium on transvaginal ultrasound and no risk factors, this patient may have been approached directly with hysteroscopy.

## Conclusions

Endometrial cancer presents with clinical symptoms like postmenopausal bleeding, which allows for early detection for better prognosis and management options. In cases of PMB with an endometrial thickness of ≤4 mm on transvaginal ultrasound, little information can be gained from endometrial sampling as the chance of getting an adequate sample is low and malignancy is rare. In addition, the presence of myomas and uterine scars can prevent proper visualization of the uterus on ultrasound. Nowadays, outpatient hysteroscopy has become a convenient and cost-effective procedure that may be done before endometrial sampling in select patients with PMB. Further research should focus on creating multiple distinctive diagnostic strategies while keeping the patient’s risk factors and characteristics in mind.

## References

[1] van Hanegem N, Breijer MC, Khan KS, Clark TJ, Burger MP, Mol BW, Timmermans A (2011). Diagnostic evaluation of the endometrium in postmenopausal bleeding: an evidence-based approach. Maturitas.

[2] Dawood NS, Peter K, Ibrar F, Dawood A (2010). Postmenopausal bleeding: causes and risk of genital tract malignancy. J Ayub Med Coll Abbottabad.

[3] American College of Obstetricians and Gynecologists (2018). The role of transvaginal ultrasonography in evaluating the endometrium of women with postmenopausal bleeding. ACOG Committee Opinion No. 734. Obstet Gynecol.

[4] Committee on Practice Bulletins—Gynecology and the Society of Gynecologic Oncology (2015). Endometrial cancer. Practice Bulletin no. 149. Obstet Gynecol.

[5] Breijer MC, Timmermans A, van Doorn HC, Mol BW, Opmeer BC (2010). Diagnostic strategies for postmenopausal bleeding. Obstet Gynecol Int.

[6] Elsandabesee D, Greenwood P (2005). The performance of Pipelle endometrial sampling in a dedicated postmenopausal bleeding clinic. J Obstet Gynaecol.

[7] Cooper NA, Barton PM, Breijer M (2014). Cost-effectiveness of diagnostic strategies for the management of abnormal uterine bleeding (heavy menstrual bleeding and post-menopausal bleeding): a decision analysis. Health Technol Assess.

